# Twelve Rules for the Year

**Published:** 1855-03

**Authors:** 


Twelve Rules for the Year.—

1. Get married—if you can; but look before you leap. Love matches are romantic, nice things to read about, but they have brimstone in them now and then, so says Ike Marvell, Esq.

2. Unite in overthrowing the fashion which translates civility into love.

3. Go to church at least once a week.

4. Whenever you see a lecture advertised, set the evening upon which it is to be delivered apart for reading fifteen pages of a good book.

5. Circulate no scandal.

6. Avoid all kinds of spirits.

7. If in the theatre, or other public place of amusement, do not level your opera glasses at strangers.

8. Never notice the clothing of persons attending divine worship, nor stand in front of the house of God after the service.

9. Never ask another man what his business is—where he is going to—where he came from—when he left—when he intends to go back, or the number of his dollars. You may inquire as to the state of his health and that of his parents, sisters and brothers—but venture no farther.

10. Defend the innocent, help the poor, and cultivate a spirit of friendship among all your acquaintances.

11. Never speak disparagingly of women, and endeavor to conquer all your prejudices. Believe all persons to be sincere in the religion which they profess.

12. Be economical, but not parsimonious nor niggardly. Make good use of your dollars, but not idols. Live within your means, and never borrow money in anticipation of your salary.

---

There are about 300 students in the medical department of the University of Nashville, (Tenn.,) instead of 241, as formerly announced.

